# Inhibition of angiotensin-induced aortic aneurysm by metformin in apolipoprotein E–deficient mice

**DOI:** 10.1016/j.jvssci.2020.11.031

**Published:** 2021-03-03

**Authors:** Anne Kunath, Jon Unosson, Malou Friederich-Persson, Niclas Bjarnegård, Mediha Becirovic-Agic, Martin Björck, Kevin Mani, Anders Wanhainen, Dick Wågsäter

**Affiliations:** a Department of Medical Cell Biology, Uppsala University, Uppsala, Sweden; b Section of Vascular Surgery, Department of Surgical Sciences, Uppsala University, Uppsala, Sweden; c Division of Drug Research, Department of Medical and Health Sciences, Linköping University, Linköping, Sweden; d Division of Cardiovascular Medicine, Department of Medical and Health Sciences, Linköping University, Linköping, Sweden

**Keywords:** Abdominal aortic aneurysm, Inflammation, Leukocytes, Metformin, T cells

## Abstract

**Objective:**

Metformin is associated with a reduced incidence and growth of abdominal aortic aneurysms (AAAs). The aim of the present study was to investigate the inhibitory effects of metformin on AAA development and possible underlying mechanisms in experimentally induced AAAs in mice, along with the possible synergistic effects of metformin and imatinib.

**Methods:**

Angiotensin II was used to induce AAAs in apolipoprotein E knockout (*ApoE*^*−/−*^) mice for 28 days. The mice were treated with metformin (n = 11), metformin combined with imatinib (n = 7), or vehicle (n = 12), starting 3 days before angiotensin II infusion. Ultrasound examination was used to analyze aneurysm formation. Cholesterol and blood pressure levels were measured at the start and end of the study. Gene array and quantitative polymerase chain reaction were used to analyze the changes in gene expression in the aorta. Wire myography was used to study vascular function.

**Results:**

Metformin (n = 11) suppressed the formation and progression of AAAs by 50% compared with the vehicle controls (n = 12), with no further effects from imatinib (n = 7). Metformin reduced total cholesterol and mRNA expression of *SPP1* (encoding osteopontin), *MMP12*, and the glycoprotein genes *Gpnmb* and *Clec7a*. Furthermore, metformin inhibited blood pressure increases and reduced vascular contractions, as determined by wire myography, and restored the anticontractile function of perivascular adipose tissue.

**Conclusion:**

Metformin inhibited aneurysm formation and progression and normalized vascular function in *ApoE*^*−/−*^ mice with no additional effect of imatinib. This might be mediated by the protective effects on vascular endothelial function and perivascular adipose tissue via reduced expression of genes promoting inflammation, including *SPP1*, *MMP12*, *Gpnmb*, and *Clec7a*.

**Clinical relevance:**

Retrospective studies of the effects of metformin in patients with aneurysm have so far only been performed of those with type 2 diabetes. The present study shows that metformin has effects on nondiabetic mice and revealed the mechanistic effects mediated by the drug that could also be important to study as outcomes in humans. Future clinical trials using metformin are warranted in patients without diabetes with abdominal aortic aneurysms.

**Article Highlights**

• **Type of Research:** A randomized case-control experimental study in mice
• **Key Findings:** The results from the present study add detailed mechanistic explanations for the effects of metformin in the prevention of angiotensin II-induced aneurysms. Metformin normalized vascular function, partly in perivascular adipose tissue, inhibited increases in mRNA expression of osteopontin (*SPP1*) and *MMP12* (genes involved in aortic aneurysms), and mRNA expression of *Gpnmb*- and *Clec7a*-encoding glycoproteins*,* which are expressed by various leukocytes. Metformin treatment in mice was associated with normalized blood pressure and total cholesterol levels.
• **Take Home Message:** Metformin inhibits several key targets in aneurysm development.

Abdominal aortic aneurysm (AAA) is a potentially lethal disease, predominantly affecting elderly men.^1^ AAA is characterized by a chronic inflammatory infiltrate, with degeneration of important connective tissue proteins and smooth muscle cells, resulting in a weakening of vessel walls, irreversible widening of the aorta, and, eventually, rupture. The precise molecular pathways underlying these disease processes are still poorly understood.

Studies in a variety of populations have reported a strong negative association between diabetes and AAA prevalence, growth, and rupture.^1^, ^2^, ^3^, ^4^ This might, in part, be attributable to the oral hypoglycemic drug metformin, which we, and others, have shown.^5^, ^6^, ^7^, ^8^ Metformin is the world's most commonly prescribed drug for type 2 diabetes and has been widely used for >60 years owing to an excellent safety profile, positive metabolic effects such as lowering cholesterol levels, and the ability to reduce cardiovascular events, over and above its effects on glycemic control.^2^^,^^5^^,^^9^

Metformin might modify several genes controlling AAA-associated inflammation. Downregulation of the expression of genes encoding osteoprotegerin (*OPN*) and osteopontin (*SPP1*)^6^^,^^7^^,^^10^^,^^11^ and reduction of macrophage infiltration in the vessel wall.^5^^,^^6^^,^^12^ A recent study showed that metformin inhibits the activation of the PI3K/AKT/mTOR/autophagy pathway.^13^ This pathway is also mediated by the tyrosine kinase inhibitor imatinib. Our previous study of imatinib treatment in AAA development revealed that T cells were the predominant leukocyte inhibited in the disease,^14^ rather than macrophages, as is the case with metformin. We, therefore, hypothesized that combination therapy would be more beneficial than single treatment. Finally, metformin has been shown to inhibit the production and activity of a range of matrix-degrading enzymes, such as matrix metalloproteinase (MMP)-9 in vitro and in animal experiments.^5^^,^^6^^,^^15^

The aim of the present study was to investigate the effects of metformin on AAA growth in a murine aneurysm model and possible underlying mechanisms by assessing vascular function using wire myography, global molecular targets using array analysis, and blood pressure, cholesterol levels, and levels of systemic inflammatory factors. We also assessed whether combination therapy with metformin and imatinib resulted in additional inhibition of AAA growth.

## Methods

### AAA model

The local ethical committee in Linköping, Sweden, provided ethical approval for the present study. Experiments were conducted in 8- to 12-week-old nondiabetic male apolipoprotein E knockout (*ApoE*^*−/−*^) mice, in accordance with the applicable guidelines. The animal care complied with the “Guide for the Care and Use of Laboratory Animals” (Institute of Laboratory Animal Resources, Commission on Life Sciences, National Research Council. Washington, DC: National Academy Press; 1996). The mice were randomly divided into four groups of 8 to 18 mice with twice as many mice allocated to the angiotensin II (AngII) groups: (1) vehicle controls; (2) vehicle with metformin treatment; (3) AngII treatment; and (4) AngII with metformin treatment. The AngII- and AngII plus metformin-treated groups received 1000 ng/kg/min AngII (Sigma Aldrich, St Louis, Mo) by chronic infusion via mini-osmotic pumps (model 1004; Alzet, Cupertino, Calif) for 28 days. In contrast, the vehicle group received 0.9% NaCl. The metformin treatment group received the drug at a dose of 100 mg/kg/d in normal drinking water for 3 days before the start of AngII treatment and for the full 28 days of AngII administration in accordance with previous studies.^6^^,^^11^^,^^12^ The mice were treated with buprenorphine (Temgesic; 0.1 mg/kg) twice daily for 48 hours postoperatively. The operated mice were checked daily for any signs of pain or disability. All the mice were fed normal chow, and water was provided ad libitum, throughout the study. The sample size was calculated to identify changes of 50% between the groups with a power of 80% and an α of 0.05.

In a second experiment, performed using a method similar to that described in the previous paragraph, the mice had received imatinib (7 mg/kg/d) for 3 days before AngII administration, as described previously,^14^ combined with metformin (100 mg/kg/d) administrated via gavage (n = 7) or vehicle (n = 10) as control for 20 days. For practical reasons and because AAA development had been established, the study time was decreased to 20 days from 28 days. No side effects of imatinib were observed during this time.

The AngII model is associated with increased blood pressure, and the use of metformin might decrease blood pressure. Blood pressure measurements were performed using a tail cuff sphygmomanometer, as previously described, to investigate whether metformin affected the blood pressure in this model.^16^ Using a standardized protocol, the aortas were divided in one half where the AAA had developed (suprarenal) or at the same location where the AAA had occurred, with the two halves used for RNA and wire myography analysis, respectively.

### Determination of AAA growth by ultrasound examination

At the end of the experiment, the mice were anesthetized with isoflurane, 2% to 4% in medical grade air, and placed in supine position on a heated examination table. A two-dimensional ultrasound biomicroscope (Vevo2100; Fujifilm VisualSonics Inc, Toronto, Ontario, Canada) equipped with a 50-MHz linear transducer (model MS 700) was used for scanning. The transducer was moved slightly in the cranial and caudal directions to judge whether any regional arterial dilatation was present along the abdominal aorta. The main anatomic landmarks were the aortic bifurcation and branches of the cephalic and mesenteric arteries. In the long-axis view, the best possible zoomed image and cine-loop (B-mode; 100 frames) were saved from the infrarenal and supracephalic segment of the aorta. B-mode images with color Doppler were saved to more accurately identify any luminal thrombus formation within the aneurysm sack. Cine-loops of the aorta at the supracephalic, paravisceral, and infrarenal levels were replayed in Vevo LAB, version 3.1.0 (Fujifilm VisualSonics), and frozen at end diastole, for determination of the diastolic diameter. At least two cardiac cycles were selected for diameter measurement at each aortic site. The calipers were placed perpendicular to the vessel at the near and far wall, respectively, in accordance with the leading edge-to-leading edge principle. The sensitivity of the method is >90%.

### Measurement of cholesterol and glucose

Total cholesterol, high-density lipoprotein (HDL), and glucose were measured twice within the study: before metformin treatment before surgery and at termination after surgery. Blood samples were taken from the tail and analyzed using the cholesterol, HDL, and glucose triple strips for CardioChek (PTS Diagnostics, Whitestown, Ind).

### Array data

To screen for genes affected by metformin treatment, total RNA was extracted from the aortas of ApoE^−/−^ vehicle control mice (n = 5), *ApoE*^*−/−*^ infused with AngII (n = 8), and *ApoE*^*−/−*^ infused with AngII and treated with metformin (n = 6), using TRIzol (Thermo Fisher Scientific, Waltham, Mass) and the RNeasy mini kit (Qiagen, Hilden, Germany). RNA quality was evaluated using the Agilent 2100 Bioanalyzer system (Agilent Technologies Inc, Palo Alto, Calif). From each sample, 200 ng of total RNA was used to generate amplified and biotinylated sense-strand cDNA from the entire expressed genome in accordance with the GeneChip WT PLUS reagent kit user manual (Thermo Fisher Scientific). Clariom D mouse transcriptome arrays (Thermo Fisher Scientific) were hybridized for 16 hours in a 45°C incubator at 60 rpm. In accordance with the Clariom D expression wash, stain and scan manual, the arrays were then washed and stained using the Fluidics Station 450 (Thermo Fisher Scientific) and scanned using the GeneChip Scanner 3000 7G (Thermo Fisher Scientific). The raw data were standardized to gene level in the transcription analysis console, version 4.0.1.36 (provided by Thermo Fisher Scientific), using the signal space transformation robust multiarray average method. Gene array analysis was performed by the Array and Analysis Facility, Uppsala University, Uppsala, Sweden.

### RNA isolation and quantitative real-time polymerase chain reaction analysis

To validate the gene array results, RNA was reverse transcribed with random primers and Superscript III (Invitrogen, Carlsbad, Calif) in accordance with the manufacturer's instructions and analyzed with real-time polymerase chain reaction (PCR). Each PCR used the standard curve method in a fluorescent temperature cycler. cDNA (1.0 ng) was amplified in a 20-μL PCR using the TaqMan Universal PCR Mastermix (Applied Biosystems, Foster City, Calif) in 96-well fast plates on a 7500 fast real-time PCR sequence detector (Applied Biosystems) in duplicate. The following probes (Thermo Fisher Scientific) were used: *CD68* (Mm03047343_m1), *Mmp12* (Mm00500554_m1), *Clec7a* (Mm01183349_m1), *Gpnmb* (Mm01328587_m1), and *Spp1* (Mm00436767_m1). The results were normalized to the values of *Tbp* (Mm00446973_m1).

### Vascular reactivity

Mouse aortic rings from the suprarenal and infrarenal regions from the vehicle control (n = 6), AngII (n = 6), and AngII with metformin treatment (n = 6) groups were evaluated for vascular reactivity using wire myography, as previously described.^17^ In brief, abdominal aortic rings were mounted on 200-μm pins in a wire myograph (Danish Myotechnology, Aarhus, Denmark). Vascular function was evaluated as cumulative concentration response curves from 1 × 10^−9^ to 1 × 10^−4.5^ mol/L noradrenaline (contraction), acetylcholine (endothelium-dependent relaxation from preconstriction to 70%-80% of a high potassium physiologic saline solution level), and sodium nitroprusside (endothelium-independent relaxation from preconstriction to 70%-80% of the potassium physiologic saline solution level). Vessels were mounted with or without the presence of perivascular adipose tissue (PVAT) after dissection under a microscope. Acute effects on vascular contraction were investigated on clean vessels from nonstimulated control mice after 30 minutes of incubation with 10 μM metformin (n = 3) or vehicle (n = 5).

### Analysis of chemokine expression in blood

The plasma samples were measured using the Bio-Plex Pro mouse chemokine panel 33-Plex (Bio-Rad Laboratories, Inc, Hercules, Calif) in accordance with the manufacturer's recommendations. An automatic magnetic washer (Magpix; Luminex Corp, Austin, Tex) was used during implementation of the assay. The 96-well microtiter plate was measured using a Luminex 200 system (Luminex Corp). All concentrations are given in pictograms per milliliter.

### Statistical analysis

Statistical analyses were performed using Prism, version 7 (GraphPad Software, La Jolla, Calif). Data are reported as the mean ± standard deviation, unless stated otherwise. Differences between the groups were analyzed using the two-tailed unpaired *t* test and one-way analysis of variance. *P* values <.05 were considered statistically significant. For vascular reactivity, values are presented as the mean ± standard error of the mean. Curves were fitted using nonlinear regression and an effective concentration of 50%. Maximal responses were evaluated using one-way analysis of variance adjusted with the Fisher least significant difference test.

## Results

### Metformin inhibition of experimental aneurysm formation in mice

A total of 54 mice were used in the initial experiment, focusing on the effects of metformin. Of the 54 mice, 17 were allocated to the AngII metformin group, 18 to the AngII vehicle group, 11 to the control metformin group, and 8 to the control vehicle group. A total of 12 mice had died (22.2%) during the AngII infusion of aortic rupture, 6 in each group (*P* = NS), and 2 were excluded for other health reasons (ie, tooth problems and constipation). No significant differences were found in the mice that had died in relation to the site of rupture (thoracic/abdominal), which had occurred in the abdominal region in four of the six mice in both groups. One half of the dead mice had belonged to the AngII plus metformin-treated group. All data from the mice that had died or had been euthanized before the experimental endpoint were excluded. Thus, the data from 40 mice were used in the initial study. The body weight gain at the end of the experimental period did not differ between the groups (data not shown).

**Fig 1:**
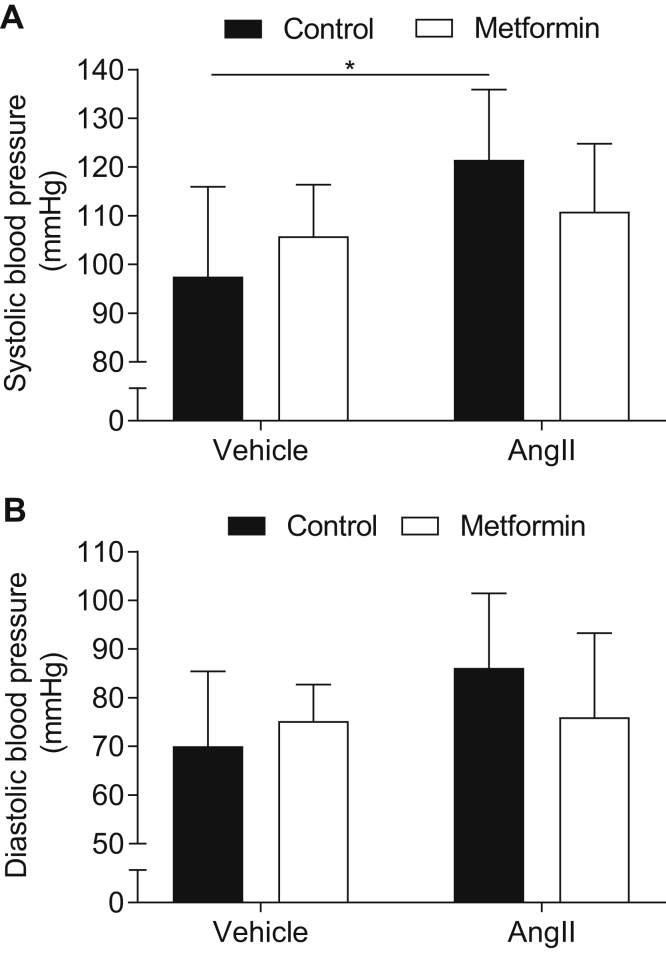
Metformin counteracts angiotensin II (AngII)-induced blood pressure (*BP*) increase in mice. Effects of metformin on systolic BP **(A)** and diastolic BP **(B)** in AngII-infused (apolipoprotein E knockout [ApoE^−/−^]) mice (n = 8-12 in each group). Data presented as the mean ± standard deviation and analyzed using analysis of variance adjusted with the Fisher least significant difference test (∗*P* < .05).

AngII infusion caused a 26% increase in systolic blood pressure compared with vehicle (saline) infusion in the *ApoE*^−/−^ mice (*P* < .05). In contrast, treatment with metformin in AngII-infused mice did not result in any significant increase in blood pressure (Fig 1). A nonsignificant trend was found in the metformin-reduced systolic and diastolic blood pressure in the AngII-infused mice.

**Fig 2:**
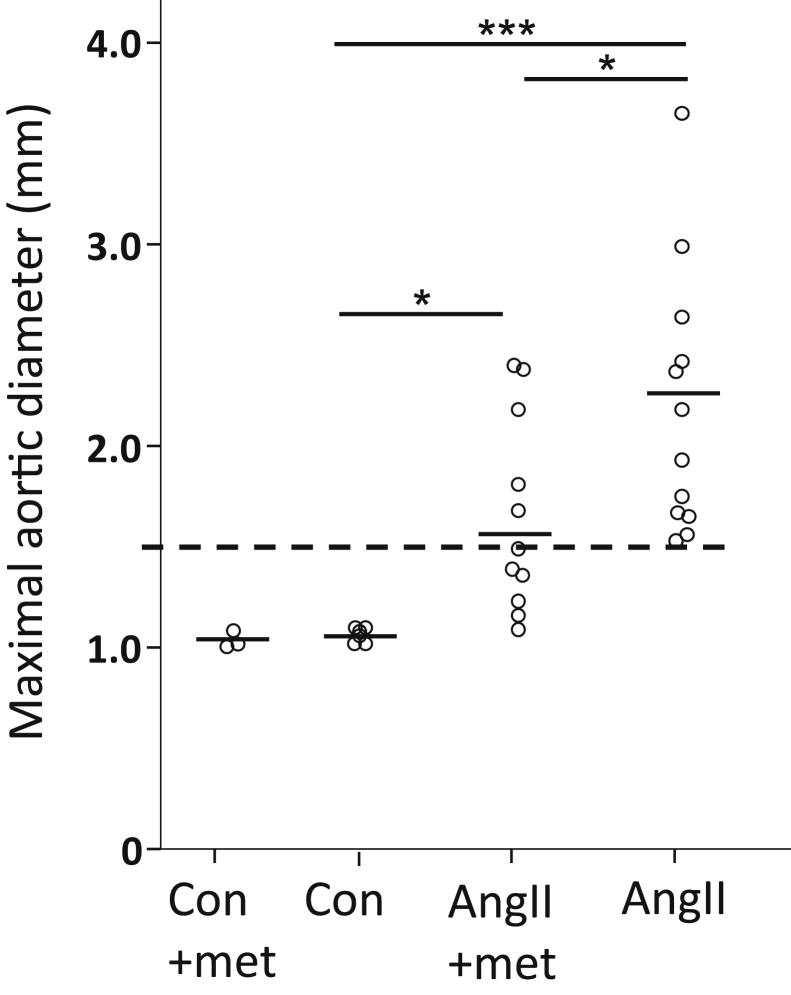
Maximum diameter of suprarenal aortas of mice at day 28 measured by ultrasound examination in non–angiotensin II (AngII)–infused mice (control [*con*], n = 6; control plus metformin [*con+met*], n = 3), mice infused with AngII with (n = 11) and without (n = 12) metformin (*met*) treatment. Data presented as individual measurements with the mean and analyzed using analysis of variance adjusted using the Fisher least significant difference test (∗*P* < .05; ∗∗∗*P* < .001). *Dashed line* indicates threshold of 50% increase in aortic diameter from that of the control mice.

In the AngII plus metformin-treated mice, only 5 of 11 (45%) had developed an AAA, with a 45% average enlargement relative to non–AngII-infused control mice. In contrast, all AngII/non–metformin-treated mice had developed an AAA, with a mean average enlargement of 120% (Fig 2).

In a second experiment, the AngII-infused mice were treated with imatinib and metformin to determine any potential effects of the combination of the two drugs' signals through the PI3K/AKT/mTOR/autophagy pathway. Adding the tyrosine kinase inhibitor imatinib to metformin treatment did not further affect AAA incidence or growth. The aortic diameter was 1.7 ± 0.47 mm in the imatinib- and metformin-treated AngII-infused animals and 1.6 ± 0.31 mm in the AngII-infused control group (*P* = NS). Of the 7 mice treated with imatinib and metformin, 3 (43%) had died of rupture or dissection, and of the 13 mice in the control group, 4 (31%) had died of rupture or dissection (*P* = NS).

**Fig 3:**
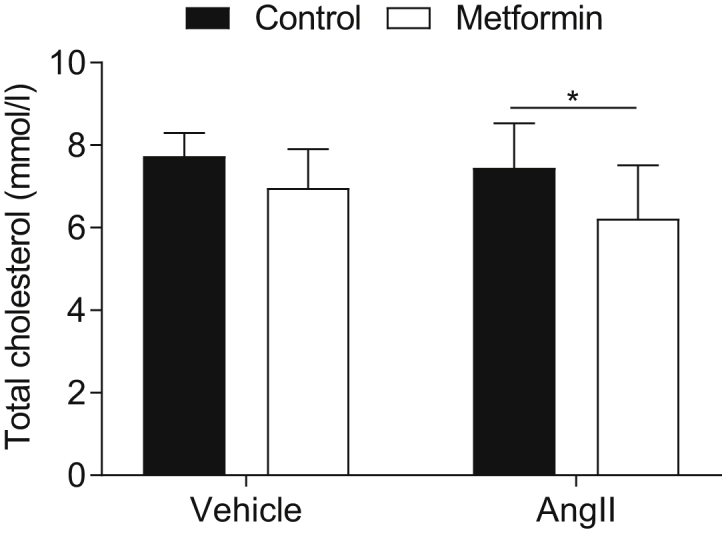
Total cholesterol at the end of the observation period (n = 7-10 in each group). Data presented as the mean ± standard deviation and analyzed using unpaired two-sample *t* tests (∗*P* < .05).

Metformin treatment significantly (*P* < .05) decreased the blood levels of total cholesterol by 20% in the AngII-treated mice (Fig 3). No influence was observed in the non–fasting blood HDL or glucose levels among the four groups (data not shown).

### Metformin normalized or improved vascular function in experimental AAA in mice

**Fig 4:**
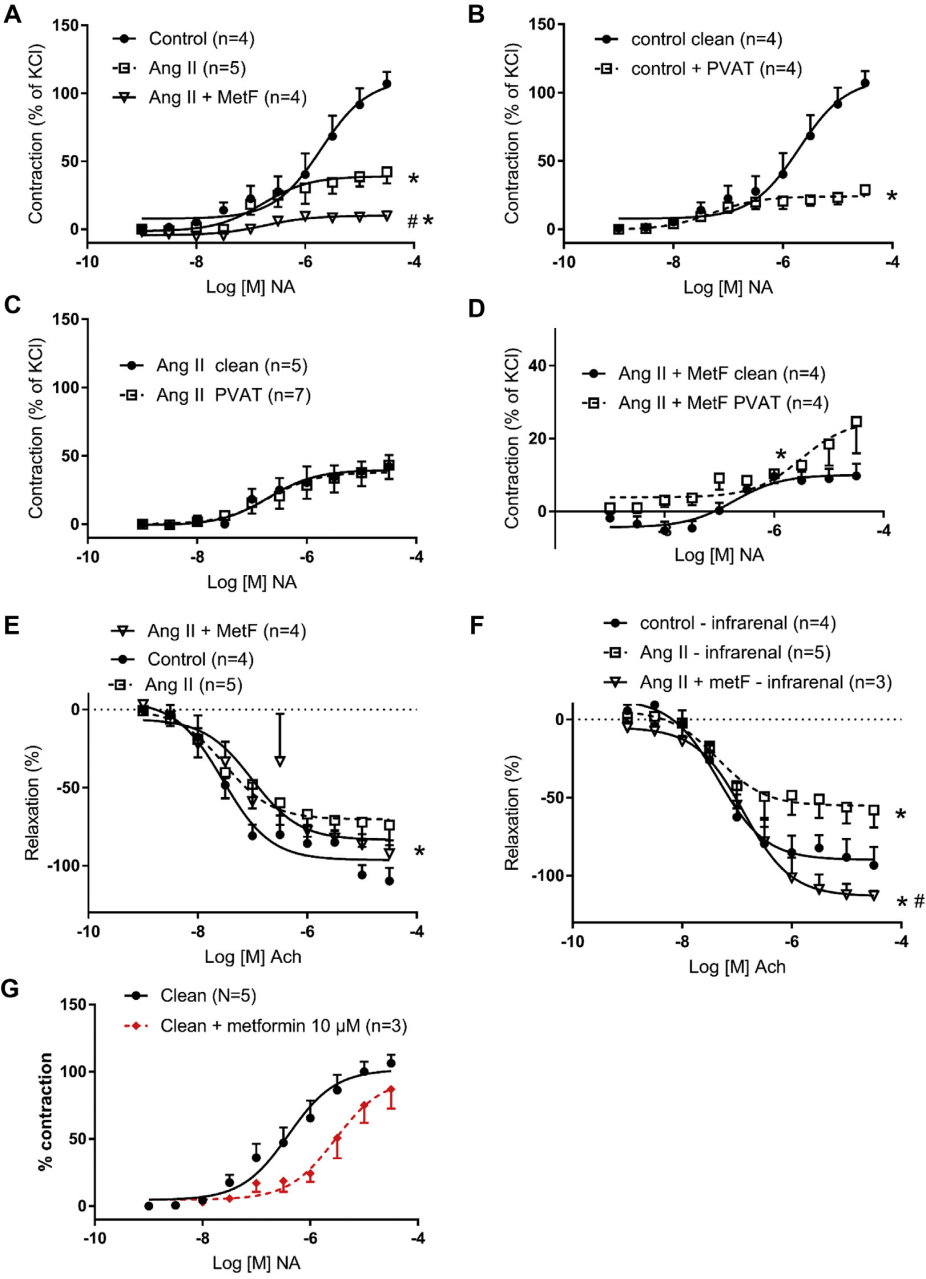
Vascular function in abdominal aortic vessels (suprarenal in **A-E** and **G**; infrarenal in **F**). **A,** Contraction response to noradrenaline (*NA*) was reduced in angiotensin II (*Ang II*)-treated mice and further reduced in hypertensive mice treated with metformin (*metF*). **B,** Perivascular adipose tissue (PVAT) exerted an anticontractile effect in control mice. **C,** PVAT from Ang II-treated mice did not exert an anticontractile effect on vessels, which was restored in metF-treated mice **(D)**. Endothelial-dependent relaxation was reduced in Ang II-treated mice **(E)** and improved in infrarenal vessels from metF-treated mice **(F)**. **G,** Acute incubation with metF on clean aortic vessels reduced the contraction response to NA. Data presented as the mean ± standard error of the mean; curves were fitted by nonlinear regression; and the effective concentration 50% and maximal concentration were evaluated using one-way analysis of variance (n = 6 in each group: **A-F**; ∗*P* < .05 vs control; #*P* < .05 vs Ang II-treated mice or metF-treated mince). **D,** A different scale was used on the Y-axis compared with the other panels. *Clean,* Vessels devoid of PVAT; *PVAT,* vessels with PVAT surrounding the vessel.

Vascular contraction in the absence of PVAT was reduced in the AngII-treated mice and further reduced in hypertensive mice treated with metformin (Fig 4, *A*). In the control mice, PVAT was protective with an anticontractile effect on the vessels (Fig 4, *B*). In contrast, PVAT from the AngII-treated mice was without effect (Fig 4, *C*). However, the anticontractile effect of PVAT was restored in the metformin-treated mice (Fig 4, *D*). No significant difference was found in the anticontractile effect between the metformin-treated AngII-infused mice and those not treated with metformin. Endothelial-dependent relaxation was reduced in the AngII-treated mice, in the suprarenal and infrarenal vessels both (Fig 4, *E* and *F*). In the infrarenal vessels, metformin treatment resulted in normalized endothelial-dependent relaxation (Fig 4, *E* and *F*). A reduced vascular contraction was also observed after acute incubation (aortas removed from wild-type nontreated mice) with metformin (Fig 4, *G*).

### Metformin reduced expression of inflammatory genes in mouse aortas with experimental AAA

**Fig 5:**
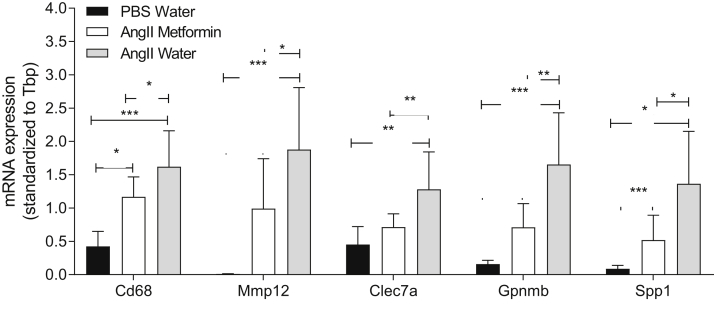
Gene expression analysis with real-time polymerase chain reaction (PCR; n = 7-12 in each group). Data presented as the mean ± standard deviation and analyzed using one-way analysis of variance adjusted with Fisher's least significant difference test (∗*P* < .05; ∗∗*P* < .01; ∗∗∗*P* < .001).

Whole transcriptome gene array analysis was applied to discover new targets involved in the development of AAAs that might be affected by metformin treatment in AngII-treated mice. Only 18 genes were found to be significantly changed by metformin treatment (Table), indicating specific drug targets. However, correction for multiple testing removed the statistical significance. Therefore, real-time PCR was used to validate eight of the candidate targets found by gene array analysis, which revealed that *CD68*, *Mmp12*, *Clec7a*, *Gpnmb*, and *Spp1* were significantly reduced by metformin treatment in AngII-infused mice (Fig 5). Three genes that were not altered in the gene array analysis remained nonsignificant on PCR validation.

**Table:** Altered genes (analyzed using whole transcriptome arrays) in mouse aortas from angiotensin II-infused mice treated with or without metformin

Gene	FC^a^	*P* value
*Ighv7-3*	−6.1	.043
*Spp1* (osteopontin)	−4.1	.001
*Atp6v0d2*	−4.0	<.001
*Gpnmb*	−4.0	<.001
*Mmp12*	−3.1	.004
*Alb*	−2.9	.034
*Clec7a*	−2.8	.008
*Mup20*	−2.7	.034
*Ms4a7*	−2.6	.026
*CD68*	−2.4	.027
*Trem2*	−2.3	<.001
*Col6a5*	−2.3	.004
*Rnu1b1*	−2.2	.015
*Mup-psp20*	−2.1	.036
*Rnu1b1*	−2.1	.020
*Rnu1b2*	−2.1	.020
*Rnu1b6*	−2.0	.024
*Hhip*	6.6	.003

*FC,* Fold change.

a FC in gene expression between metformin (n = 6) and no metformin (n = 8) mice treated with angiotensin II (*P* value computed using the Student *t* test); the results were not significant when adjusted using the false discovery rate.

**Fig 6:**
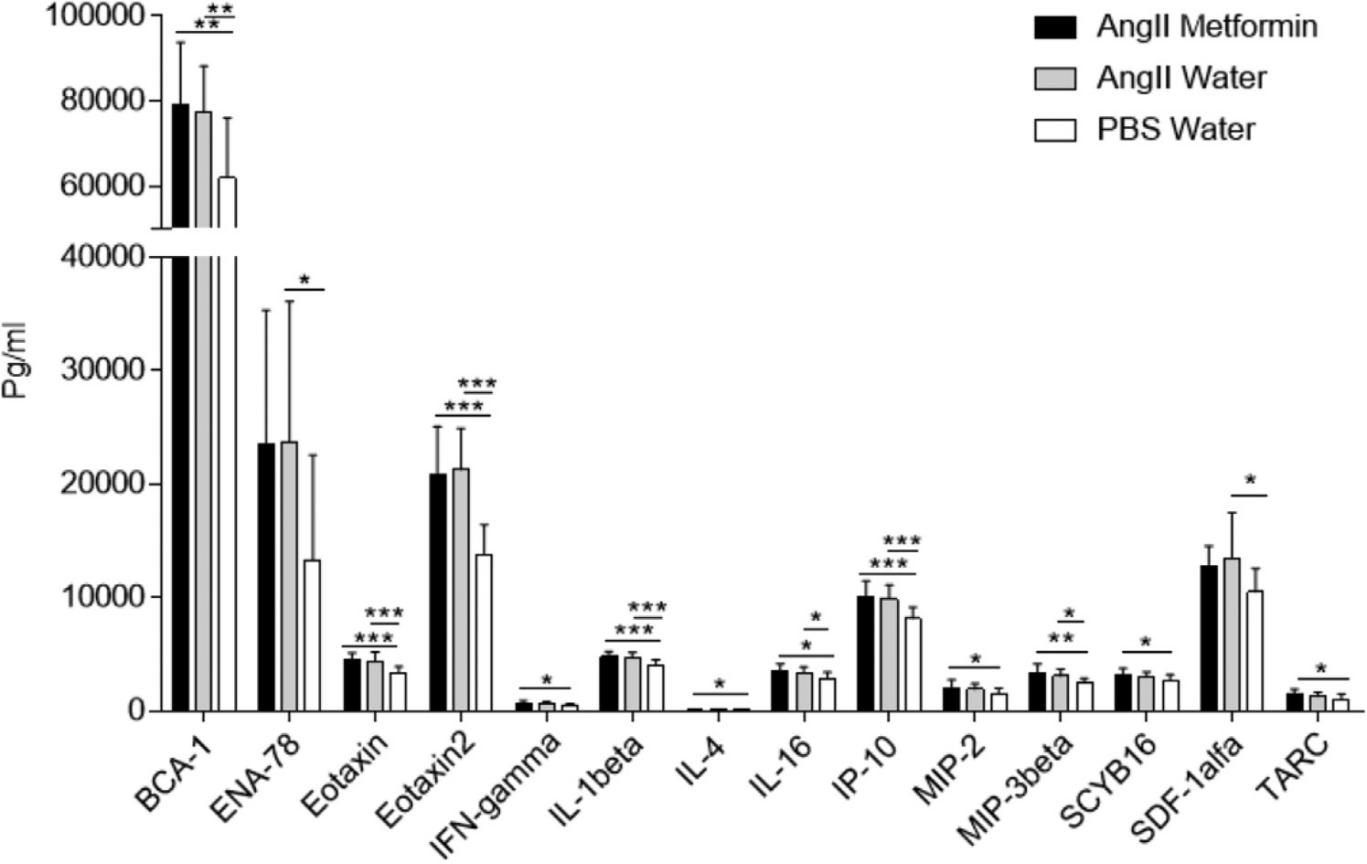
Cytokine profile in controls (n = 8), angiotensin II (AngII)-treated mice (n = 12), and AngII plus metformin-treated mice (n = 11) . Data presented as the mean ± standard deviation and analyzed using one-way analysis of variance adjusted with the Fisher least significant difference test (∗*P* < .05; ∗∗*P* < .01; ∗∗∗*P* < .001).

Because aneurysm disease is driven by inflammation, blood from the different groups of mice was analyzed for changes in a panel of cytokines to identify any potential targets of metformin action. Of 33 cytokines, 14 were altered in the AngII-treated mice compared with the control mice (Fig 6). However, no changes were found in the expression of the factors among the AngII-treated mice, regardless of whether they had received metformin.

## Discussion

A negative association between diabetes and the prevalence of AAAs was first described by Lederle et al^1^ in 1997 in the large ADAM (aneurysm detection and management) study. Since then, several reports have confirmed this observation. It has also been shown that diabetes is associated with slower AAA growth.^18^, ^19^, ^20^ The results from recent observational studies have suggested that the protective effect of diabetes on AAA is more likely a result of the antidiabetic treatment, specifically metformin.^3^^,^^4^^,^^8^

In agreement with previous studies using both elastase and AngII, we found that metformin reduced disease onset and attenuated AngII-induced AAA growth in metformin-treated nondiabetic mice. Thus, metformin limited the initiation and progression of murine AAAs, when administered continuously during aneurysm induction and enlargement.^5^^,^^6^^,^^12^ Death by rupture was not prevented by metformin in our study, which could potentially be explained because the angiotensin model starts with dissection before aneurysm development. Combining ruptured aortas with those defined as an AAA did not result in any protective effects from metformin in the AngII-infused mice. However, in the study by Yang et al,^12^ death by dissection was inhibited by metformin.

The results from the present study have also demonstrated functional effects of metformin on the aorta. Vascular contraction was reduced in AAAs and was further reduced by metformin administration. Reduced vascular contraction was also observed after acute incubation with metformin, highlighting a direct effect on the vessels. Metformin reduced the endothelial dysfunction often demonstrated in untreated hypertensive models.^21^ In a related pathology, diet-induced obesity, it was shown that metformin improved endothelial function.^22^ Although a reduction of contractile function with metformin treatment might seem counterintuitive and could be taken to indicate a worsening of vascular function, we propose instead that it is a mechanism to protect the vascular wall and, consequently, reduces AAA development. This mechanism appears to be both dependent and independent of the aortic PVAT. In accordance with previous studies, the control mice displayed an anticontractile effect of PVAT (ie, a desensitized response to a contractile hormone).^17^ The protective function of PVAT was first described by Soltis and Cassis^23^ as reducing the contractile response of aortic vessels, later denoted as the anticontractile effect of PVAT. Other studies have highlighted that the mechanism underlying the protective effect of PVAT is complex and multifactorial.^24^ The protective function of PVAT was lost in the untreated hypertensive mice in the present study. This is in accordance with the results from other studies in which the protective effect of PVAT was lost in the presence of hypertension, obesity, and diabetes.^25^^,^^26^ However, in our study, PVAT function was restored by metformin administration. To the best of our knowledge, the present study was the first to demonstrate protective vascular effects from metformin that are partly mediated by PVAT in an AngII-infused AAA model. PVAT is important for the normal physiology of the aorta. We previously found that PVAT surrounding AAA is pathologic and contains a local source of inflammatory leukocytes and proteases.^27^ The reduced macrophage content in metformin-treated aortas might hypothetically be explained by the effects of metformin in PVAT, which normalizes vascular function and inhibits adventitial inflammation and degradation of the vessel wall. We could not rule out that a part of the mechanisms might be due to endothelial-dependent relaxation. The exact role of metformin on PVAT requires exploration in more detailed studies.

The reduction of vascular contraction by metformin would also serve to reduce total peripheral resistance and, thereby, lower blood pressure. In the present study, the effects of metformin on systolic and diastolic blood pressure was investigated in an AngII-infused *ApoE*^−/−^ mouse model of AAA. Compared with the vehicle mice, AngII increased systolic blood pressure by ∼24 mm Hg during the 28-day study period. This was expected because AngII is an active hormone of the renin-angiotensin system known to play an important role in the development of hypertension. In the present study, a nonsignificant trend was found that metformin reduced systolic and diastolic blood pressure in AngII-infused mice.

To screen for possible novel genes involved in the mechanisms of metformin, a gene array analysis of the aortas was performed. Eighteen genes significantly changed by metformin treatment were discovered, of which several are novel in respect to metformin effect. None of the genes remained significant after false discovery rate adjustment. However, this was probably a type II error caused by the low number of mice. A validation using real-time PCR on the same genes was performed, and five of these genes—*CD68* (macrophages), *Mmp12* and *Spp1* (osteopontin), and *Clec7a* and *Gpnmb* (glycoproteins)—were significantly reduced by metformin treatment. The genes that were nonsignificant in the gene array analysis remained nonsignificant in the real-time PCR validation. One of the altered genes, *Spp1*, encodes a protein with a role both in the proinflammatory extracellular matrix and as a circulating proinflammatory cytokine. The connection between AAA and osteopontin was first described by Golledge et al,^10^ who found an association between osteopontin serum and tissue concentrations with human AAAs and suggested osteopontin as a useful biomarker for AAA presence and growth.

Degradation of elastin and collagen in AAA is caused by a family of endopeptidases called matrix metalloproteinases (MMPs). Several members of the MMP family have been implicated in AAA pathogenesis.^28^^,^^29^ Previous studies have demonstrated an important role for MMP-12 in various inflammatory and degenerative conditions. Curci et al^30^ suggested a unique role for MMP-12 in human AAA pathogenesis. Longo et al^28^ supported the hypothesis, reporting findings that AAA induction in *Mmp12*^*−/−*^ mice led to a small increase in aortic diameter compared with the increase seen in wild-type mice. In contrast, Pyo et al^31^ could not confirm that aneurysmal degeneration was suppressed by *Mmp12* deficiency. These two studies had used different mouse models. Longo et al^28^ had used the CaCl_2_ model, and Pyo et al^31^ had used the elastase-infused AAA mouse model. With the AngII-infused aneurysm model, we found a reduction of *Mmp12* expression throughout metformin administration in our study, further evidence for inhibition of inflammation by metformin. Since the depletion of either MMP-12 or osteoprotegerin has been associated with ruptured AAAs, one might suspect that the lowered expression of these two genes by metformin in our study would inhibit rupture. However, metformin only partially decreased the expression of these two targets compared with their expression in non–metformin-treated AngII mice and not to the levels in the control mice and, therefore, not sufficient to inhibit aortic rupture.

*Clec7a* (involved in surface pattern recognition) and *Gpnmb* (involved in negative regulation of cell proliferation) are two genes previously found to be upregulated in elastase-induced AAA in rabbits.^32^ In our study, they were upregulated by angiotensin in mice. Both these genes encode glycoproteins that are expressed on dendritic cells, macrophages, and B cells, which are all found in AAAs. Their roles in aneurysm disease are as yet unexplored.

Because metformin is an inhibitor of mitochondrial function, the changes in some of the gene expression might be related to changes in metabolic shift and cell respiration. This was not investigated in the present study but would be interesting to explore.

Aneurysm disease involves inflammation, and, because previous studies have shown that metformin inhibits inflammation, we included the analysis of several inflammatory factors in the blood to determine whether the inhibition of aneurysm by metformin was associated with any of these factors. Metformin did not influence any of the 33 factors in the blood. Our results in mice are somewhat in agreement with a previous study by Wang et al^33^ of humans, indicating a more local effect on the inflammatory process in the aorta.

The present study had several limitations that should be addressed. Only male mice were used, limiting the results to this gender. Most research of AAAs in animals is performed using males to keep the variation low and limit the number of animals needed. Another plausible limitation is that we were unable to measure the aortic diameter for 10 of the control aortas for technical reasons. However, macroscopic observation of those aortas not measured by ultrasound examination did not reveal any pathologic findings. Because the most important comparisons were performed between the angiotensin groups treated with or without metformin, we do not believe this affected the interpretation of the results. Ruptured aortas were not included in the gene expression analysis because we could not control for RNA degradation. When including the ruptured aortas to compare any protective effects of metformin in the AngII-infused mice, metformin did not result in any total significant protective effects in the present study. Because the AngII model is initiated with a dissection, we believe that ruptured and intact AAAs should be analyzed separately. We also did not analyze the aortic diameter in the ruptured aortas because they had occurred at different points throughout the study period, it would have been difficult to compare them with those that fulfilled the study period. With respect to myography analysis, the few number of aortas limited the significant differences between the groups (ie, Fig 4, *C* and *D*). Finally, the differences in gene expression between the aortas probably reflected the changes in cell composition, predominantly macrophages, that metformin inhibit.

## Conclusion

In the present experimental study of AngII-induced AAA mice, we identified several potential mechanisms whereby metformin could prevent AAA formation by targeting vascular function and highlighting the importance of PVAT. Combining metformin with imatinib did not affect inhibition of AAA development. A study in which AngII infusion starts before metformin treatment would be of interest to investigate its effects on an already developed AAA. Further clinical studies, preferably randomized controlled trials of patients without diabetes with small AAAs, are warranted to assess the potential for metformin as a medical therapy for patients with small AAAs.

## Author contributions

Conception and design: AK, MB, AW, DW

Analysis and interpretation: AK, JU, MFP, NB, MBA, MB, KM, AW, DW

Data collection: AK, MFP, NB, MBA, DW

Writing the article: AK, JU, MFP, MB, AW, DW

Critical revision of the article: AK, JU, MFP, NB, MBA, MB, KM, AW, DW

Final approval of the article: AK, JU, MF, NB, MBA, MB, KM, AW, DW

Statistical analysis: AK, MFP, NB, MBA, DW

Obtained funding: AW, DW, AK

Overall responsibility: DW
